# (E)-N-(2-(3, 5-Dimethoxystyryl) phenyl) furan-2-carboxamide (BK3C231) induces cytoprotection in CCD18-Co human colon fibroblast cells through Nrf2/ARE pathway activation

**DOI:** 10.1038/s41598-021-83163-7

**Published:** 2021-02-26

**Authors:** Huan Huan Tan, Noel Francis Thomas, Salmaan Hussain Inayat-Hussain, Kok Meng Chan

**Affiliations:** 1 Center for Toxicology and Health Risk Studies, Faculty of Health Sciences, Universiti Kebangsaan Malaysia, 50300 Kuala Lumpur, Malaysia; 2 Methodist College Kuala Lumpur, 50470 Kuala Lumpur, Malaysia; 3 Product Stewardship and Toxicology, Group Health, Safety, Security and Environment, Petroliam Nasional Berhad (PETRONAS), 50088 Kuala Lumpur, Malaysia; 4 Department of Environmental Health Sciences, Yale School of Public Health, 60 College St, New Haven, CT 06250 USA

**Keywords:** Biochemistry, Cell biology, Molecular biology

## Abstract

Cytoprotection involving the nuclear factor erythroid 2-related factor 2 (Nrf2)/antioxidant response element (ARE) signaling pathway is an important preventive strategy for normal cells against carcinogenesis. In our previous study, the chemopreventive potential of (E)-N-(2-(3, 5-Dimethoxystyryl) phenyl) furan-2-carboxamide (BK3C231) has been elucidated through its cytoprotective effects against DNA and mitochondrial damages in the human colon fibroblast CCD-18Co cell model. Therefore this study aimed to investigate the molecular mechanisms underlying BK3C231-induced cytoprotection and the involvement of the Nrf2/ARE pathway. The cells were pretreated with BK3C231 before exposure to carcinogen 4-nitroquinoline N-oxide (4NQO). BK3C231 increased the protein expression and activity of cytoprotective enzymes namely NAD(P)H:quinone oxidoreductase 1 (NQO1), glutathione S-transferase (GST) and heme oxygenase-1 (HO-1), as well as restoring the expression of glutamate-cysteine ligase catalytic subunit (GCLC) back to the basal level. Furthermore, dissociation of Nrf2 from its inhibitory protein, Keap1, and ARE promoter activity were upregulated in cells pretreated with BK3C231. Taken together, our findings suggest that BK3C231 exerts cytoprotection by activating the Nrf2 signaling pathway which leads to ARE-mediated upregulation of cytoprotective proteins. This study provides new mechanistic insights into BK3C231 chemopreventive activities and highlights the importance of stilbene derivatives upon development as a potential chemopreventive agent.

## Introduction

Cytoprotective mechanisms are orchestrated whenever cellular homeostasis is disrupted upon exposure to endogenous or exogenous chemical insults. Upregulation of cytoprotective protein expression through specific redox-sensitive transcriptor factor activation is crucial to ensure cell survival and more importantly, to protect the cells against carcinogenesis^1^. Transcription factor Nrf2 primarily mediates transcriptional activation through ARE which is found in the promoter region of many cytoprotective genes namely NQO1, GST, HO-1, and GCLC essential for maintaining cellular redox homeostasis^2,3^.

Stilbenes are secondary metabolites produced as plant defense compounds with a wide range of health-beneficial effects^4^. The stilbene and its many derivatives have been reported to possess cancer chemopreventive activities by inhibiting cellular events associated with tumour initiation, promotion, and progression which are the three general stages of chemical carcinogenesis^5,6^. Hence, they are promising compounds that fulfill the search for new cancer chemopreventive agents to replace the conventionally used cancer chemopreventive agents with adverse side effects.

Our group has reported the upregulation of NQO1 by BK3C231 in human embryonic hepatocytes, WRL-68 cells^7^. Our previous study further elucidated the chemopreventive and cytoprotective effects of BKC231 whereby it protects CCD-18Co cells against 4NQO-induced DNA and mitochondrial damages^8^. The aim of this study was to investigate the molecular mechanisms underlying BK3C231-induced cytoprotection and the role of BK3C231 in the Nrf2/ARE signaling pathway. As the induction of cytoprotective enzymes serves as a pivotal mechanistic tool in cancer prevention, we anticipate that our findings in this study will expand the horizon of stilbene and its synthetic derivatives in chemoprevention research.

## Results

### BK3C231 increased activity of cytoprotective enzymes in 4NQO-treated CCD-18Co cells

**Figure 1:**
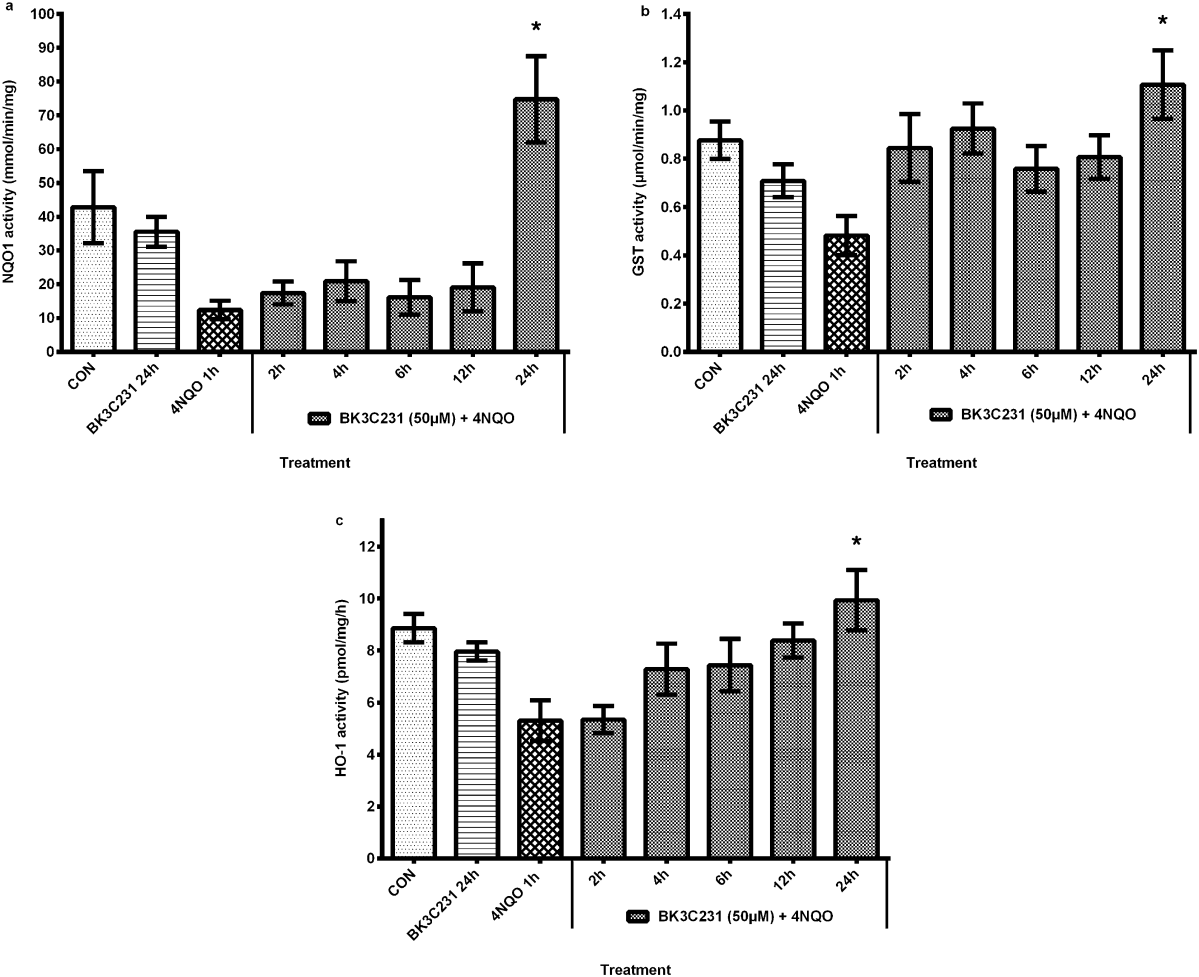
Effect of BK3C231 pretreatment on the activity of cytoprotective enzymes namely NQO1 (**a**), GST (**b**), and HO-1 (**c**). The enzymatic activity of cytoprotective enzymes was observed at basal level in untreated control cells (CON) and cells treated only with BK3C231 (BK3C231 24 h). However, in cells treated only with 4NQO (4NQO 1 h), there was a decrease in the activity of cytoprotective enzymes as compared to that of untreated control cells. Pretreatment of cells with BK3C231 before 4NQO exposure increased the activity of cytoprotective enzymes, significantly at 24 h as compared to that of cells treated with 4NQO only. Treated whole cell lysates were subjected to spectrophotometric and microplate reader data analysis as mentioned in “Results” to determine the activity of NQO1, GST and HO-1. Each data point was expressed as mean ± SEM from at least three independent experimental replicates. * p < 0.05 against 4NQO 1 h.

The enzymatic activity of cytoprotective enzymes namely NQO1, GST, and HO-1 was observed at basal level in untreated control cells and cells treated only with BK3C231 (Fig. 1). However, in cells treated only with 4NQO, there was a decrease in activity level up to 0.7-fold for NQO1, 0.5-fold for GST, and 0.4-fold for HO-1 over untreated control.

Pretreatment of cells with BK3C231 for 24 h before 4NQO exposure significantly increased NQO1 activity up to fivefold at 74.76 ± 12.76 nmol/min/mg as compared to that of 4NQO-treated cells at 12.41 ± 2.72 nmol/min/mg. The enhancement of the NQO1 activity level by BK3C231 was almost 2 times higher than the basal level with a value of 42.81 ± 10.65 nmol/min/mg (Fig. 1a). As for GST activity, our study demonstrated a 1.3-fold increase from 0.48 ± 0.08 µmol/min/mg in 4NQO-treated cells to 1.11 ± 0.14 µmol/min/mg in BK3C231-pretreated cells for 24 h. BK3C231 also enhanced GST activity level by up to 0.2-fold increase over basal level at 0.88 ± 0.08 µmol/min/mg (Fig. 1b).

Moreover, HO-1 activity level significantly increased up to 0.9-fold from 5.30 ± 0.78 pmol/mg/h in 4NQO-treated cells to 9.93 ± 1.16 pmol/mg/h upon BK3C231 pretreatment for 24 h. HO-1 activity in BK3C231-pretreated cells at 12 h was also restored to the basal level which was at 8.87 ± 0.55 pmol/mg/h (Fig. 1c).

### BK3C231 elevated protein expression of cytoprotective enzymes in 4NQO-treated CCD-18Co cells

**Figure 2:**
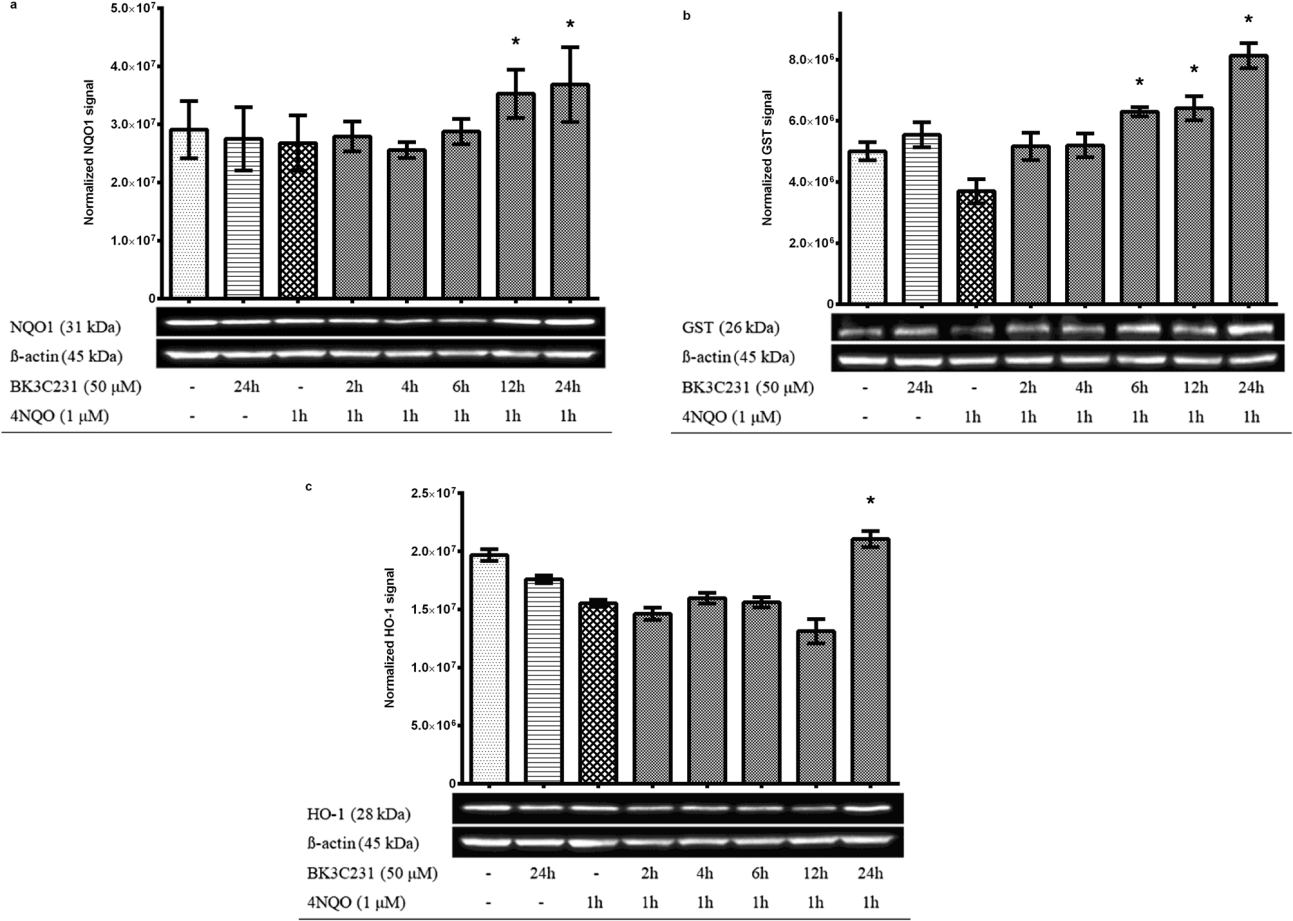
Effect of BK3C231 pretreatment on the protein expression of cytoprotective enzymes namely NQO1 (**a**), GST (**b**), and HO-1 (**c**). Basal-level constitutive expressions of NQO1, GST and HO-1 were observed in untreated control cells and cells treated only with BK3C231. In cells treated only with 4NQO, the expression level of cytoprotective enzymes decreased as compared to that of untreated control. Pretreatment of cells with BK3C231 before 4NQO exposure significantly increased the protein expression of cytoprotective enzymes at 12 h and 24 h for NQO1; 6 h, 12 h and 24 h for GST; and 24 h for HO-1 as compared to that of cells treated with 4NQO only. Treated whole cell lysates were subjected to immunoblotting analysis to determine the protein expression level of NQO1, GST, and HO-1. Each data point was expressed as mean ± SEM from at least three independent experimental replicates. *p < 0.05 against 4NQO 1 h. The blots displayed in the figure are cropped. Full-length blots are presented in Supplementary Figure S2.

Basal-level constitutive expressions of NQO1, GST, HO-1 and GCLC were observed in untreated control cells and cells treated only with BK3C231 as shown in Fig. 2 and Supplementary Fig. S1. In cells treated only with 4NQO, the expression level of cytoprotective enzymes decreased by 8% for NQO1, 32.8% for GST, 21.1% for HO-1 and 24.3% for GCLC, over untreated control.

Interestingly, BK3C231 pretreatment induced up to a 0.4-fold increase of NQO1 expression significantly at 12 h and 24 h in 4NQO-stimulated cells as compared to that of cells treated with 4NQO only. NQO1 expression in BK3C231-pretreated cells was also 26.6% higher than the basal level observed in untreated control cells (Fig. 2a and Supplementary Fig. S2a). Cells pretreated with BK3C231 demonstrated up to 1.2-fold increase of GST expression significantly from 6 to 24 h over 4NQO-treated cells. BK3C231 also enhanced GST expression by 47.7% over the basal level (Fig. 2b and Supplementary Fig. S2b).

Other than that, HO-1 expression was elevated significantly up to 0.4-fold upon BK3C231 pretreatment for 24 h before 4NQO exposure as compared to that of 4NQO-treated cells and increased by 7% over the basal level (Fig. 2c and Supplementary Fig. S2c). GCLC expression in BK3C231-pretreated cells increased up to 0.3-fold particularly at 12 h and 24 as compared to that of 4NQO-treated cells and recovered to the basal level (Supplementary Fig. S1a,b). Since the induction of cytoprotective enzymes is mainly regulated by the transcription factor Nrf2, we further examined the role of BK3C231 in the activation of Nrf2.

### BK3C231 promoted Nrf2 protein expression level, reduced Keap1 protein expression level and induced Nrf2 activation through dissociation from Keap1

**Figure 3:**
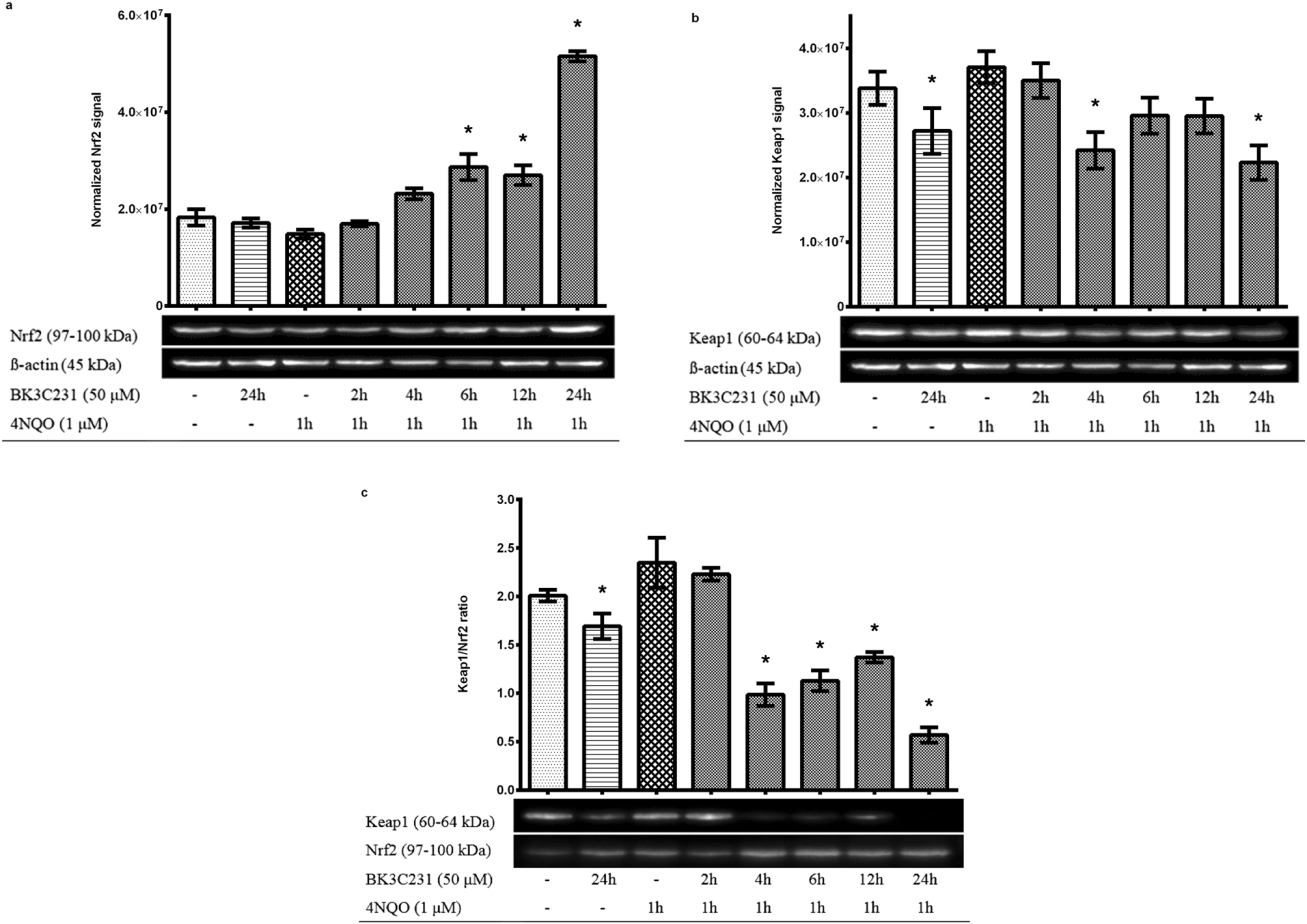
Effect of BK3C231 pretreatment on Nrf2 (**a**) and Keap1 (**b**) protein expression levels and dissociation level of Nrf2 from Keap1 (**c**). (**a**, **b**) Constitutive expressions of Nrf2 and Keap1 at the basal level were observed in untreated control cells. 4NQO reduced Nrf2 expression and increased Keap1 expression over untreated control. Cells treated only with BK3C231 showed a significant reduction of Keap1 expression over 4NQO-treated cells whereas Nrf2 expression remained at basal level. BK3C231 pretreatment for 6 h, 12 h, and 24 h significantly increased Nrf2 expression over 4NQO-treated cells. Keap1 expression was significantly reduced in cells pretreated with BK3C231 at 4 h and 24 as compared to that of 4NQO-treated cells. (**c**) The Keap1/Nrf2 ratio was increased in 4NQO-treated cells over untreated control. BK3C231 pretreatment from as early as 4 h till 24 h significantly reduced Keap1/Nrf2 ratio over 4NQO-treated cells and untreated control, thereby inducing dissociation of Nrf2 and Keap1 leading to Nrf2 activation. Treated whole cell lysates were subjected to immunoblotting analysis to determine the expression level of Nrf2 and Keap1. Treated whole cell lysates were subjected to co-immunoprecipitation as mentioned in “Results” and immunoblotting analysis to determine the Keap1 to Nrf2 ratio. Each data point was expressed as mean ± SEM from at least three independent experimental replicates. *p < 0.05 against 4NQO 1 h. The blots displayed in the figure are cropped. Full-length blots are presented in Supplementary Fig. S3.

Constitutive expressions of Nrf2 and Keap1 at the basal level were observed in untreated control cells. 4NQO reduced Nrf2 expression by 18.8% and increased Keap1 expression by 9.6% over untreated control. However, cells treated only with BK3C231 showed a significant reduction of Keap1 expression by 26.6% over 4NQO-treated cells whereas Nrf2 expression increased by 15.4% over 4NQO-treated cells and remained around the basal level (Fig. 3a,b and Supplementary Fig. S3a,b).

Interestingly, BK3C231 pretreatment for 6 h, 12 h, and 24 h significantly increased Nrf2 expression up to 2.5-fold over 4NQO-treated cells. Nrf2 expression was also enhanced up to 1.8-fold over basal level upon BK3C231 pretreatment for 24 h (Fig. 3a). Other than that, Keap1 expression was significantly reduced by up to 39.8% in cells pretreated with BK3C231 at 4 h and 24 as compared to that of 4NQO-treated cells (Fig. 3b). BK3C231 pretreatment also decreased Keap1 expression by 34% over untreated control cells.

Furthermore, to determine the role of BK3C231 in Nrf2 activation, the dissociation level of Nrf2 from its inhibitor protein Keap1 was evaluated using co-immunoprecipitation assay to pull-down Nrf2. The Keap1/Nrf2 ratio was increased by 0.2-fold in 4NQO-treated cells over untreated control (Fig. 3c and Supplementary Fig. S3c). BK3C231 pretreatment from as early as 4 h till 24 h significantly reduced Keap1/Nrf2 ratio up to 0.8-fold over 4NQO-treated cells and untreated control, thereby inducing dissociation of Nrf2 and Keap1 leading to Nrf2 activation.

### Cytoprotective enzymes were upregulated through an increase in ARE promoter activity by activated Nrf2

**Figure 4:**
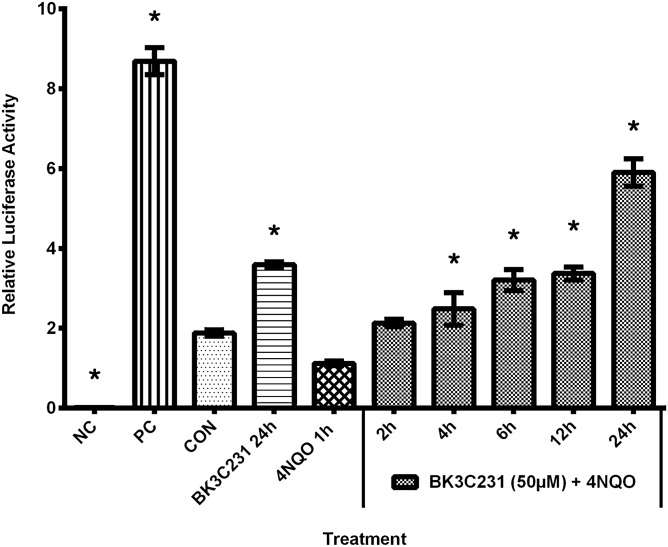
Effect of BK3C231 pretreatment on ARE promoter activity. The relative luciferase activity which indicates ARE promoter activity was observed at basal level in untreated control cells (CON). Treatment of cells with 4NQO only (4NQO 1 h) decreased ARE promoter activity over untreated control. However, in cells treated only with BK3C231 (BK3C231 24 h), relative luciferase activity significantly increased over 4NQO-treated cells (4NQO 1 h). BK3C231 treatment alone also enhanced relative luciferase activity over the basal level in the untreated control (CON). BK3C231 pretreatment significantly increased relative luciferase activity from 4 h till 24 h over 4NQO-treated cells (4NQO 1 h). Cells were transfected with ARE reporter construct and pretreated with BK3C231 before 4NQO exposure as mentioned in “Results”. Subsequently, the dual-luciferase assay was performed to determine the firefly and Renilla luciferase activities. Cells transfected with Cignal negative control and positive control reporter constructs were used as the negative control (NC) and positive control (PC) for this assay. Each data point was expressed as mean ± SEM from at least three independent experimental replicates. *p < 0.05 against 4NQO 1 h.

ARE promoter activity as indicated by the relative luciferase activity was observed at basal level in untreated control cells with a value of 1.88 ± 0.08 (Fig. 4). 4NQO decreased ARE promoter activity up to 0.4-fold at 1.12 ± 0.06 over untreated control. However, in cells treated only with BK3C231, relative luciferase activity significantly increased up to 2.2-fold at 3.59 ± 0.07 over 4NQO-treated cells. BK3C231 treatment alone also enhanced relative luciferase activity up to 0.9-fold over the basal level in the untreated control. BK3C231 pretreatment significantly increased relative luciferase activity up to 4.3-fold at 2.48 ± 0.41 for 4 h, 3.20 ± 0.26 for 6 h, 3.37 ± 0.16 for 12 h, and 5.90 ± 0.35 for 24 h over 4NQO-treated cells.

## Discussion

Our previous study has reported that BK3C231 protected CCD-18Co cells against 4NQO-induced DNA and mitochondrial damages by decreasing DNA strand breaks and micronucleus formation as well as reducing the loss of mitochondrial membrane potential and cardiolipin^8^. Our previous study has also demonstrated that nitrosative stress instead of oxidative stress was involved in 4NQO-induced DNA and mitochondrial damages. 4NQO induced generation of NO but not reactive oxygen species (ROS), leading to nitrosative stress instead of oxidative stress which has been reported in previous studies^9,10^. BK3C231 exerted these cytoprotective effects in CCD-18Co cells by suppressing 4NQO-induced nitrosative stress through a reduction in nitric oxide (NO) level and upregulation of glutathione (GSH) level.

Cytoprotective enzymes, also known as phase II detoxifying and antioxidant enzymes, defend and protect mammalian cells from various forms of stress, thus serving as an important target for preventing normal cells from developing diseases and malignancy such as cancer. These cytoprotective enzymes which include NQO1, GCLC, GST, and HO-1 are involved in an elaborate system of detoxification and elimination of carcinogens, as well as maintaining cellular redox status against electrophilic and oxidative stresses^11–14^.

In the present study, we demonstrated that pretreatment of cells with BK3C231 before 4NQO exposure increased activity and protein expression of the cytoprotective enzymes above the basal level which were otherwise reduced in cells exposed only to 4NQO. The increase in GSH level by BK3C231 as shown in our previous study is likely due to restoration of GCLC protein, which is the catalytic unit of glutamate-cysteine ligase that catalyzes the rate-limiting step in the formation of GSH, back to the basal level upon BK3C231 pretreatment. Besides, BK3C231 was able to induce NQO1 and GST which function in minimizing toxic reactive intermediates and prevent intracellular depletion of thiol pools, as well as HO-1 which is a rate-limiting enzyme that forms a potent antioxidant called bilirubin.

These inducible cytoprotective proteins require ARE-mediated transcription through activation of the redox-sensitive transcription factor Nrf2^15^. Moreover, with GST and GCLC as Nrf2 target genes, Nrf2 controls the expression of key components of GSH and therefore is established as a regulator of GSH, which is one of the most important antioxidants in the body^16–18^. Under basal condition, Nrf2 is being kept inactive in the cytoplasm by the binding of an inhibitory protein, Keap1 which constitutively promotes Nrf2 ubiquitylation and proteasomal degradation, resulting in low protein level of Nrf2 in non-stressed cells^19–22^. Keap1, also known as Kelch-like ECH associated protein 1, is the primary regulator of the Nrf2-dependent cellular antioxidant response whereby it negatively regulates Nrf2 by enhancing its rate of proteasomal degradation and altering its subcellular distribution^23,24^. Therefore, Keap1 functions as the main chemical sensor responsible for the regulation of Nrf2 activation^25^.

There have been a plethora of studies reporting activation of the Nrf2/ARE pathway in response to oxidative stress^2,13,26^, but the role of nitrosative stress in the Nrf2/ARE pathway activation has not been very well understood. Interestingly, in this study, we demonstrated that the nitrosative stress induced by 4NQO was able to reduce Nrf2 expression slightly below the basal level as well as increasing Keap1 expression and stabilizing the association between Keap1 and Nrf2. This was in contrast with a previous study which demonstrated NO-induced Keap1 inactivation leading to Nrf2 stabilization^27^. Fourquet et al.^27^ suggested that NO-induced Keap1 inactivation involves a multistep reaction process with ROS and oxygen, therefore the absence of ROS generation by 4NQO may explain the inability of NO as the sole reactive nitrogen species (RNS) to inactivate Keap1.

In response to electrophilic and oxidative stresses mainly generated by electrophiles or xenobiotics, activation of Nrf2 is mainly mediated by mechanisms promoting its dissociation from Keap1, leading to ARE-mediated expression of cytoprotective enzymes^19,25,28,29^. As a short-lived protein, Nrf2 is also activated by mechanisms leading to its stabilization in cells under stress^21,30^. In line with this, given the constitutively low Nrf2 level and short half-life of Nrf2 under basal condition, we are able to demonstrate that BK3C231 pretreatment prior to 4NQO induction increased Nrf2 protein level and decreased Keap1 level concurrently up till 24 h suggesting that BK3C231 reduced Nrf2 ubiquitylation and degradation which was promoted by Keap1 as well as inducing Keap1 inactivation. Hence, BK3C231 pretreatment leads to Nrf2 activation by enhancing Nrf2 synthesis and stabilization of Nrf2 protein which was usually transient^31^. Activation of Nrf2 in response to stress signals are most likely a result of its stabilization, mediated by mechanisms that decrease the rate of its degradation which is in line with the findings of our study.

In addition, BK3C231 induced Nrf2 activation through disruption of Keap1-Nrf2 complex as shown by a decreased amount of Keap1 that co-immunoprecipitated with Nrf2, thus leading to an increase in ARE promoter activity upon Nrf2 activation and upregulation of cytoprotective enzymes. Dissociation of Nrf2 from Keap1 is either facilitated through Nrf2 phosphorylation by activated protein kinases or interaction of cysteine residues present in Keap1 with prooxidants or electrophiles^32^. Therefore, the mechanisms underlying BK3C231-induced Nrf2 activation merits further investigation to strengthen our understanding of the cytoprotective and chemopreventive role of BK3C231.

**Figure 5:**
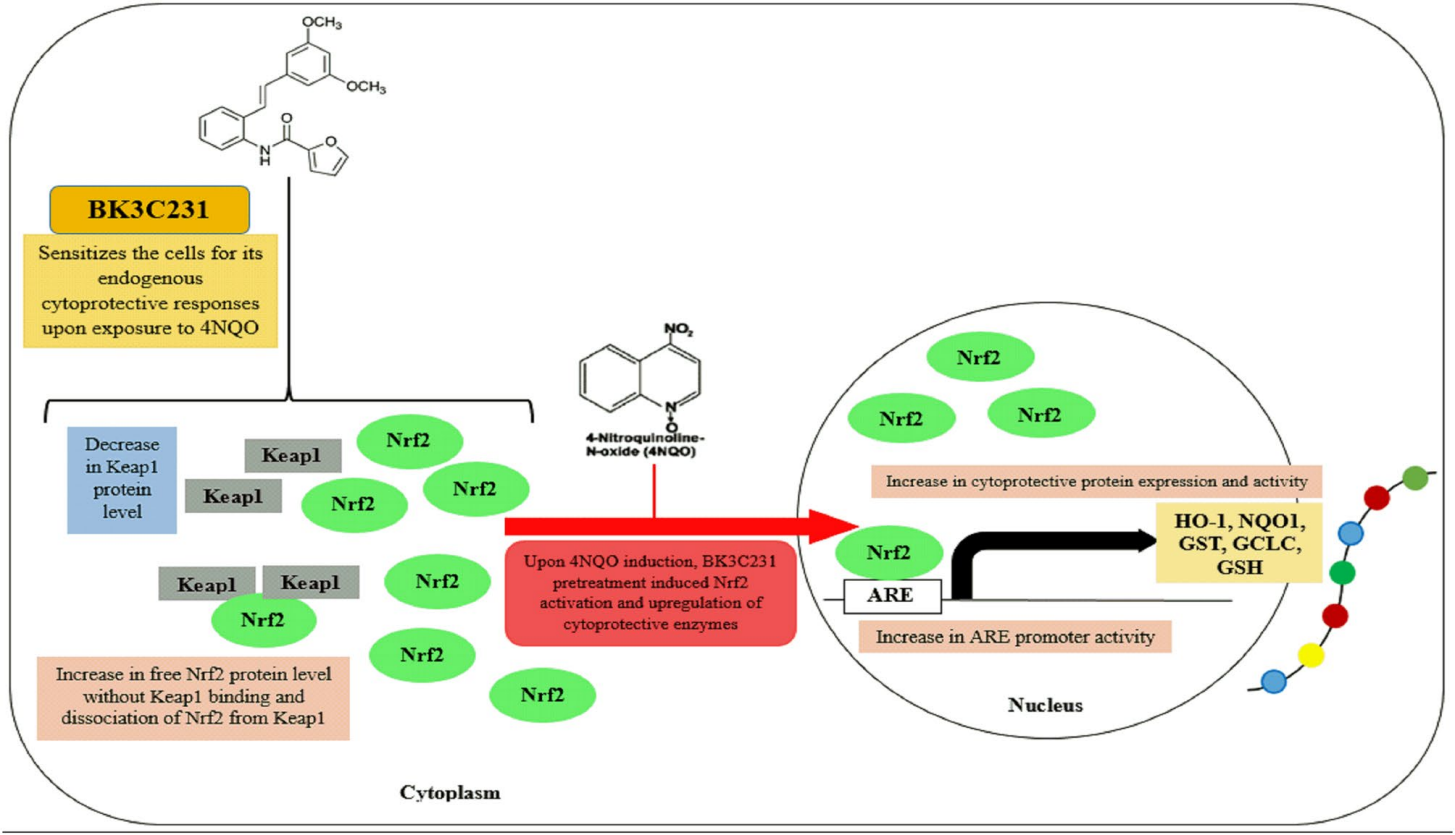
Schematic illustration representing the cytoprotective effects of BK3C231 through Nrf2 activation which leads to ARE-mediated upregulation of cytoprotecive enzymes. Upon exposure of cells to 4NQO, BK3C231 pretreatment increased protein expression and activity of the cytoprotective enzymes namely NQO1, GST and HO-1, as well as maintaining GCLC at basal level. BK3C231 pretreatment prior to 4NQO exposure induced Nrf2 activation by increasing Nrf2 protein level and decreasing Keap1 level, as well as promoting disruption of Keap1-Nrf2 complex thus leading to an increase in ARE promoter activity upon Nrf2 activation and upregulation of cytoprotective enzymes. Interestingly, reduction in Keap1 level which lead to an increase in the dissociation level of Nrf2 from Keap1 as well as ARE promoter activity were observed in cells treated only with BK3C231, hereby suggesting that BK3C231 is able to sensitize the cells for its endogenous cytoprotective responses and elevate the cytoprotective capacity of cells upon exposure to stress inducers or toxicants such as 4NQO.

Interestingly, our study has also demonstrated that in cells treated only with BK3C231, there is a significant reduction in Keap1 level which lead to an increase in the dissociation level of Nrf2 from Keap1 as well as ARE promoter activity. However, the Nrf2 level remained at basal level which explains the reason for the level of downstream cytoprotective proteins at basal level. Due to the reduction of Keap1 protein expression as well as increases in the dissociation level of Nrf2 from Keap1 and ARE promoter activity by BK3C231 under basal cell condition, BK3C231 pretreatment is able to elicit the cytoprotective effects at a rapid rate as seen in cells towards 1 h of 4NQO exposure. Therefore, this study suggests that BK3C231 is able to sensitize the cells for its endogenous cytoprotective responses. Based on our present findings, we hereby proposed a schematic diagram to illustrate the cytoprotective effects of BK3C231 through Nrf2 activation which leads to ARE-mediated upregulation of cytoprotecive enzymes (Fig. 5). Our study suggests that the cytoprotective capacity of cells is enhanced in the presence of BK3C231 upon exposure of cells to toxicants, thus rendering BK3C231 a potential chemopreventive agent.

## Conclusion

In conclusion, BK3C231 conferred cytoprotection in 4NQO-induced CCD18-Co cells through the induction of cytoprotective enzymes by activating the Nrf2/ARE pathway. We anticipate this study will accelerate the development of BK3C231 as a potential drug for chemoprevention.

## Methods

### Cell culture

CCD-18Co cell line was obtained from the American Type Culture Collection (ATCC; Manassas, VA). CCD-18Co cells were grown in Eagle’s Minimum Essential Medium (EMEM; Gibco, Grand Island, NY) supplemented with 10% fetal bovine serum (FBS; Biowest, Nuaillé, France) and 1% 100 × Antibiotic–Antimycotic solution (Nacalai Tesque, Kyoto, Japan). All cells were between passages 3–5 for all experiments and maintained at 37 °C with 5% CO_2_.

### BK3C231 and 4NQO treatment

BK3C231 was synthesized and provided by Dr. Noel Francis Thomas and Dr. Kee Chin Hui from the University of Malaya, Malaysia. Both BK3C231 and 4NQO (Sigma, St. Louis, MO) were dissolved in solvent dimethyl sulfoxide (DMSO; Fisher Scientific, Hampton, NH). The final concentration of DMSO used as a vehicle for the compounds did not exceed 0.05% (v/v). 0.05% of DMSO exerted no changes in cell viability as assessed using MTT cytotoxicity assay (Supplementary Table S1). Generally, CCD-18Co cells were plated onto cell culture dishes (60 × 15 mm) at the concentration of 5 × 10^4^ cells/ml for 24 h. Cells were pretreated with BK3C231 at 50 µM for 2 h, 4 h, 6 h, 12 h, and 24 h before 4NQO induction at 1 µM for 1 h. The control to BK3C231 used is the negative control which is the culture media only without any addition of treatments on cells. The negative control, BK3C231 treatment alone and 4NQO treatment alone were performed in parallel to the BK3C231-pretreatment conditions.

### NQO1 activity assay

This method was carried out as described previously with modifications^33^. The treated cells were trypsinized and collected by centrifugation (450×*g*/5 min at 4 °C). The resulting pellet was washed with chilled phosphate-buffered saline (PBS) and resuspended with 100 μl of homogenization buffer consisting of 25 mM Tris at pH 7.4 (Bio-Rad Laboratories, Hercules, CA), 250 mM sucrose (Sigma, St. Louis, MO), and 5 μM flavin adenine dinucleotide (FAD; Sigma, St. Louis, MO). Reaction buffer consisting of 25 mM Tris at pH 7.4 (Bio-Rad Laboratories, Hercules, CA), 1 mg/ml bovine serum albumin (BSA; Sigma, St. Louis, MO), and 200 μM nicotinamide adenine dinucleotide (NADH; Sigma, St. Louis, MO) was prepared fresh. Then, 970 μl of reaction buffer was added to the cuvette and placed in a spectrophotometer to be adjusted to zero reading. 20 μl of 2 mM dichlorophenolindophenol (DCPIP; Sigma, St. Louis, MO) was then added before the addition of 10 μl sample in the cuvette and mixed by inversion. The cuvette was immediately placed in a spectrophotometer and the absorbance was recorded at 600 nm from 0 to 1 min. Two additional aliquots of the sample were repeated to provide triplicate measurements. The DCPIP reaction was repeated using the same sample volume of 1 ml by adding 10 μl of 2 mM dicoumarol (Sigma, St. Louis, MO), instead of sample, to the mixture described above. This reaction was repeated two times to provide triplicate measurements. NQO1 activity was expressed as micromoles of reduced DCPIP per minute per milligram of protein using the DCPIP molar extinction coefficient of 21,000 and protein concentration of the sample.

### GST activity assay

This method was carried out as described previously with modifications^34^. The treated cells were lysed in lysis buffer containing 50 mM dipotassium phosphate (K_2_HPO_4_; Sigma, St. Louis, MO), 1 mM ethylenediaminetetraacetic acid (EDTA; Sigma, St. Louis, MO) at pH 6.5 and 0.1% v/v Triton X (Sigma, St. Louis, MO). The reaction mixture was prepared fresh at the proportion of 980 μl PBS at pH 6.5, 10 μl of 100 mM glutathione (GSH; Sigma, St. Louis, MO), and 10 μl of 100 mM 2,4-dinitrochlorobenzene (CDNB; Sigma, St. Louis, MO). The spectrophotometer was set at 340 nm and blank absorbance was read with 1 ml of the reaction mixture as background control. Then, 950 μl of the reaction mixture was added with 50 μl sample in a cuvette and placed immediately in the spectrophotometer. An increase in absorbance was read every 30 s for 5 min after a lag time of 1 min. GST activity was expressed as micromoles of conjugated CDNB per minute per milligram of protein using CDNB conjugate extinction coefficient of 9.6 mM^−1^ and protein concentration of the sample.

### HO-1 activity assay

This method was carried out as described previously with modifications^35^. The treated cells were resuspended in homogenization buffer containing 0.25 M sucrose (Sigma, St. Louis, MO), 20 mM Tris–HCl at pH 7.4 (Bio-Rad Laboratories, Hercules, CA), 1 mM EDTA (Sigma, St. Louis, MO), and 0.1% protease inhibitor cocktail (Roche, Mannheim, Germany). Reaction mixture consisting of 100 μl sample, 2 mg/ml biliverdin reductase from rat liver, 1 mM β-nicotinamide adenine dinucleotide phosphate (β-NADPH; Sigma, St. Louis, MO), 2 mM glucose-6-phosphate (Sigma, St. Louis, MO), 1 U glucose-6-phosphate dehydrogenase (G6PD; Sigma, St. Louis, MO) and 25 μM hemin (Sigma, St. Louis, MO) was prepared fresh and incubated at 37 °C for 1 h in the dark. The formed bilirubin was extracted with chloroform and the change in optical density at 464–530 nm was measured in a microplate reader (Multiskan Go; Thermo Fisher Scientific, Waltham, MA). HO activity was expressed as picomoles of bilirubin formed per milligram of protein per hour by using an extinction coefficient of 40 mM^-1^/cm for bilirubin in chloroform and protein concentration of the sample.

### Immunoblotting analysis

Briefly, the treated cells were lysed in radio-immunoprecipitation assay (RIPA) buffer (Sigma, St. Louis, MO) containing 1 mM dithiothreitol (DTT; Sigma, St. Louis, MO) and protease inhibitor (Roche, Mannheim, Germany). The lysates were then solubilised in 5X loading buffer and denatured at 95 °C for 5 min. Then, 15 μg of each sample was subjected to 12% SDS–polyacrylamide gel electrophoresis (SDS-PAGE) and transferred onto a polyvinylidene fluoride (PVDF) membrane. BLUeye Prestained Protein Ladder (GeneDireX, Inc., Taiwan, China) was loaded along with the samples for SDS-PAGE prior to immunoblotting. The blots were then incubated with anti-NQO1 antibody (diluted 1:1000; Abcam, Cambridge, UK), anti-GST antibody (diluted 1:1000; Abcam, Cambridge, UK), anti-GCLC antibody (diluted 1:1000; Abcam, Cambridge, UK), anti-HO-1 antibody (diluted 1:1000; Cell Signaling Technology, Danvers, MA), anti-Nrf2 antibody (diluted 1:1000; Cell Signaling Technology, Danvers, MA), anti-Keap1 antibody (diluted 1:1000; Cell Signaling Technology, Danvers, MA) and anti-beta-actin antibody (dilution 1:5000; Cell Signaling Technology, Danvers, MA); followed by anti-rabbit IgG, horseradish peroxidase (HRP)-conjugated secondary antibody (diluted 1:1000; Cell Signaling Technology, Danvers, MA) using SNAP i.d. 2.0 protein detection system (Millipore, Billerica, MA). The blots were stained with Luminata Forte Western HRP substrate (Millipore, Billerica, MA) and visualised using Fusion-FX7 gel documentation (Vilber Lourmat, Collegien, France) for enhanced chemiluminescent detection. The signal intensity was quantified relative to the loading control (beta actin) by performing densitometry using Fusion-Capt Advance software (Vilber Lourmat, Collegien, France).

### Co-immunoprecipitation of Nrf2 and Keap1

This method was carried out as described previously with modifications^29^. Briefly, 400 μg of whole-cell lysates were incubated with anti-Nrf2 antibody (diluted 1:100; Abcam, Cambridge, UK) overnight at 4 °C under gentle agitation. Then, 30 μl of protein G plus/protein A agarose suspension (Calbiochem, San Diego, CA) was added to each sample and further incubated for 3 h at 4 °C under gentle agitation. The immune complexes were collected by centrifugation (4000×*g*/5 min at 4 °C) and washed with RIPA buffer for 3 times. The resulting pellet was solubilized in 2X loading buffer and denatured at 95 °C for 5 min. Samples were then centrifuged and the supernatants were subjected to immunoblotting analysis.

### Dual-luciferase reporter assay

Generally, the cells were plated onto 24-well plates and subjected to forward transfection with ARE reporter construct (Cignal Reporter Assay kits; Qiagen, Hilden, Germany) using Lipofectamine 3000 transfection reagent (Thermo Fisher Scientific, Waltham, MA) according to manufacturer's protocol. After 48 h of transfection, cells were pretreated with 50 µM of BK3C231 for 2 h, 4 h, 6 h, 12 h, and 24 h before 4NQO induction at 1 µM for 1 h. Then, the dual-luciferase assay was performed using the Dual-Luciferase Reporter (DLR) assay system (Promega, Madison, WI) according to manufacturer’s protocol following the method as described previously with modifications^36^. Relative luciferase activities were analyzed by normalizing the ARE-mediated firefly luciferase (experimental reporter) activities to Renilla luciferase (control reporter) activities. Cells transfected with Cignal negative control and positive control reporter constructs were used as negative control and positive control for this assay.

### Statistical analysis

The data are expressed as the mean ± standard error of the mean (S.E.M.) from at least three independent experiments. The Shapiro–Wilk test was used to test data for normality. The statistical significance was evaluated using one-way ANOVA with the Tukey post hoc test used to assess the significance of differences between multiple treatment groups. Differences were considered statistically significant with a probability level of p < 0.05.

## Supplementary Information

Supplementary Information.
