# Supplementary material for: (E)-N-(2-(3, 5-Dimethoxystyryl) phenyl) furan-2-carboxamide (BK3C231) induces cytoprotection in CCD18-Co human colon fibroblast cells through Nrf2/ARE pathway activation
Source: Sci Rep. 2021 Feb 26;11:4773. doi: 10.1038/s41598-021-83163-7 (PMC7910600; doi:10.1038/s41598-021-83163-7)
Supplement: Supplementary file 1 — Supplementary Information. [file 41598_2021_83163_MOESM1_ESM.pdf]

Supplementary Information

(E)-N-(2-(3, 5-Dimethoxystyryl) phenyl) furan-2-carboxamide (BK3C231) induces cytoprotection in CCD18-Co human colon fibroblast cells through Nrf2/ARE pathway activation

Huan Huan Tan[1], Noel Francis Thomas[2], Salmaan Hussain Inayat-Hussain[3,4], Kok Meng Chan[1,*]

[1]Center for Toxicology and Health Risk Studies, Faculty of Health Sciences, Universiti Kebangsaan Malaysia, Kuala Lumpur, 50300, Malaysia.
[2]Methodist College Kuala Lumpur, Kuala Lumpur, 50470, Malaysia.
[3]Product Stewardship and Toxicology, Group Health, Safety, Security and Environment, Petroliam Nasional Berhad (PETRONAS), Kuala Lumpur, 50088, Malaysia.
[4]Department of Environmental Health Sciences, Yale School of Public Health, 60 College St, New Haven, CT 06250, USA.

[*]Corresponding author:
Chan Kok Meng, PhD
Associate Professor
Toxicology and Risk Assessment Research Group,
Centre for Health and Applied Sciences,
Faculty of Health Sciences,
Universiti Kebangsaan Malaysia
50300 Kuala Lumpur, Malaysia

Email: chan@ukm.edu.my
Tel: +603-92898118
Fax: +03-26929032

Supplementary Figure S1. Effect of BK3C231 on the expression of cytoprotective enzyme GCLC as assessed using immunoblotting analysis. (a) GCLC expression in BK3C231-pretreated cells increased at 12 h and 24 as compared to that of 4NQO-treated cells and recovered to the basal level. Each data point was expressed as mean ± SEM from at least three independent experimental replicates. The blots displayed in the figure are cropped. **(b)** Full-length blots showing GCLC protein band and loading control, β-actin protein band run on the same blot.

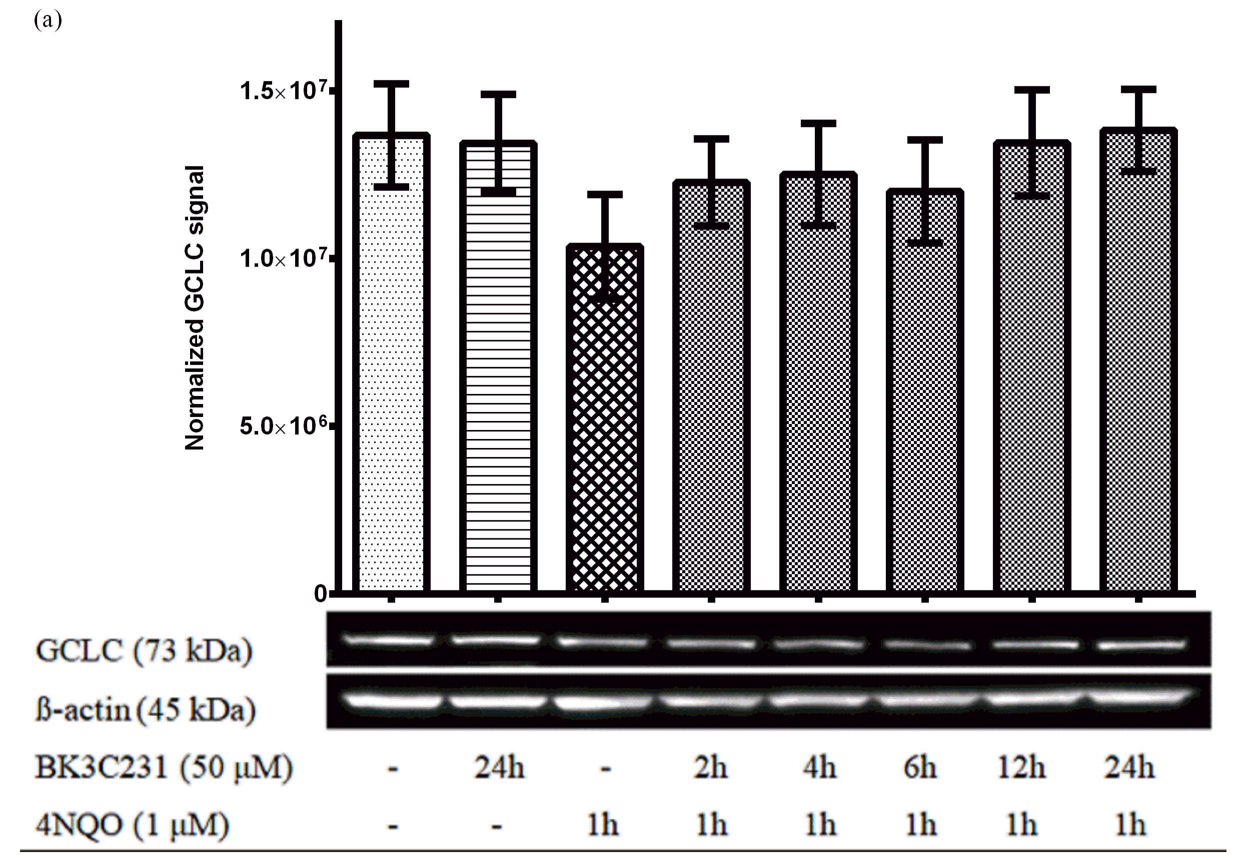

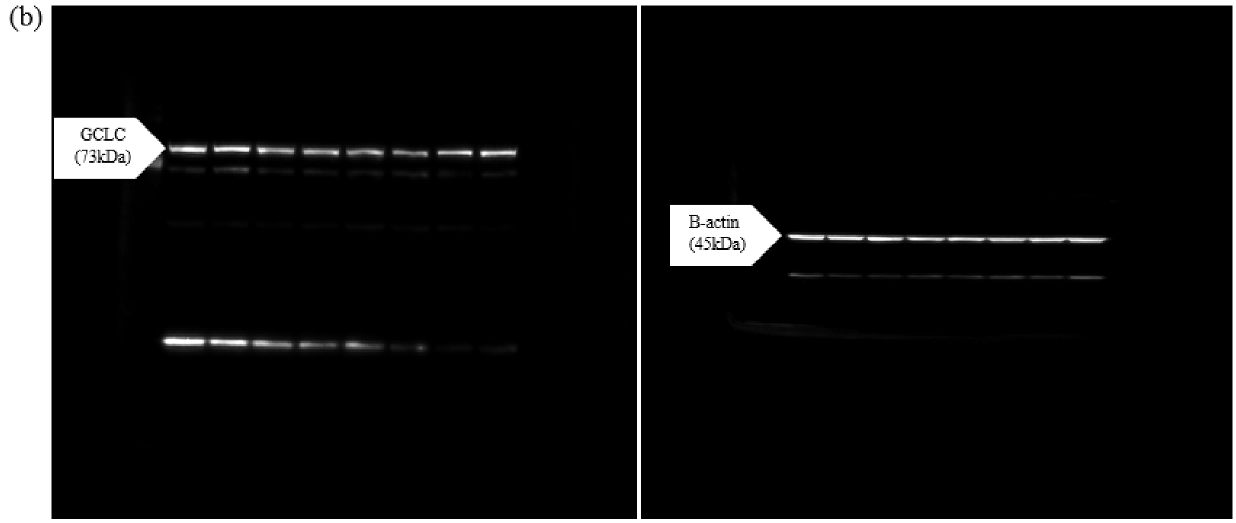

Supplementary Figure S2. The expression of cytoprotective enzymes was elevated by BK3C231 as assessed using immunoblotting analysis. (a) Full-length blots showing NQO1 protein band and loading control, β-actin protein band run on the same blot. **(b)** Full-length blots showing GST protein band and loading control, β-actin protein band run on the same blot. **(c)** Full-length blots showing HO-1 protein band and loading control, β-actin protein band run on the same blot.

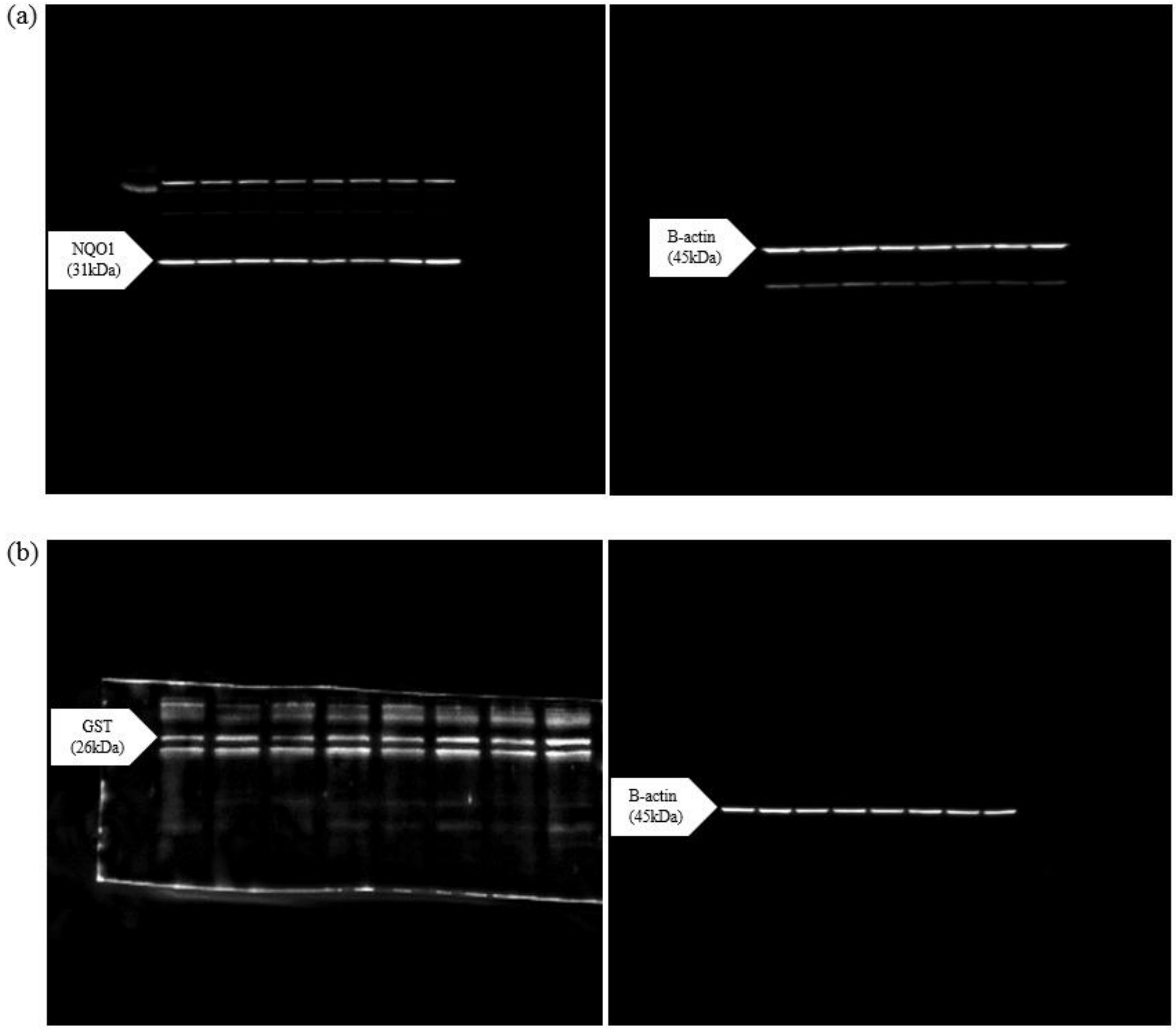

(c)

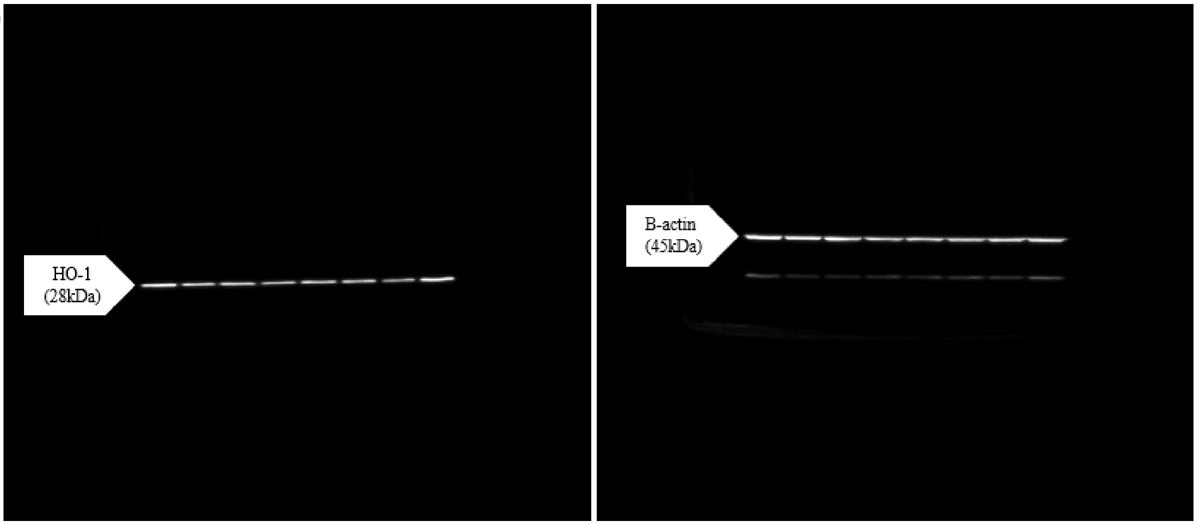

Supplementary Figure S3. Nrf2 expression level and activation were induced by BK3C231 as assessed using immunoblotting analysis. (a) Full-length blots showing Nrf2 protein band and loading control, β-actin protein band run on the same blot. **(b)** Full-length blots showing Keap1 protein band and loading control, β-actin protein band run on the same blot. **The dissociation level of Nrf2 from Keap1 was increased by BK3C231 as assessed using co-immunoprecipitation and immunoblotting assays to determine the Keap1 to Nrf2 ratio. (c)** Full-length blots showing Keap1 and Nrf2 protein bands run on the same blot.

(a)

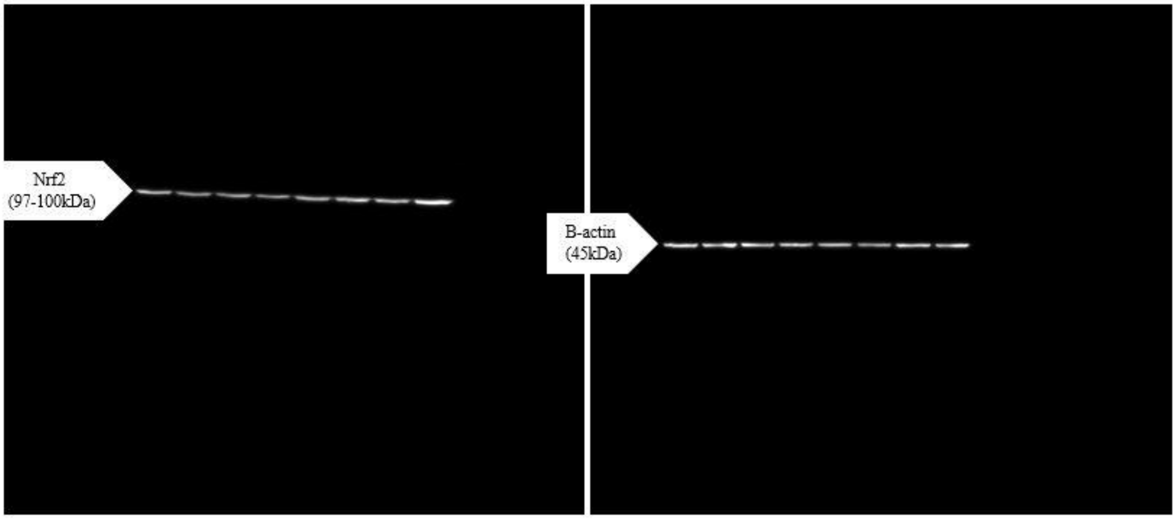

(b)

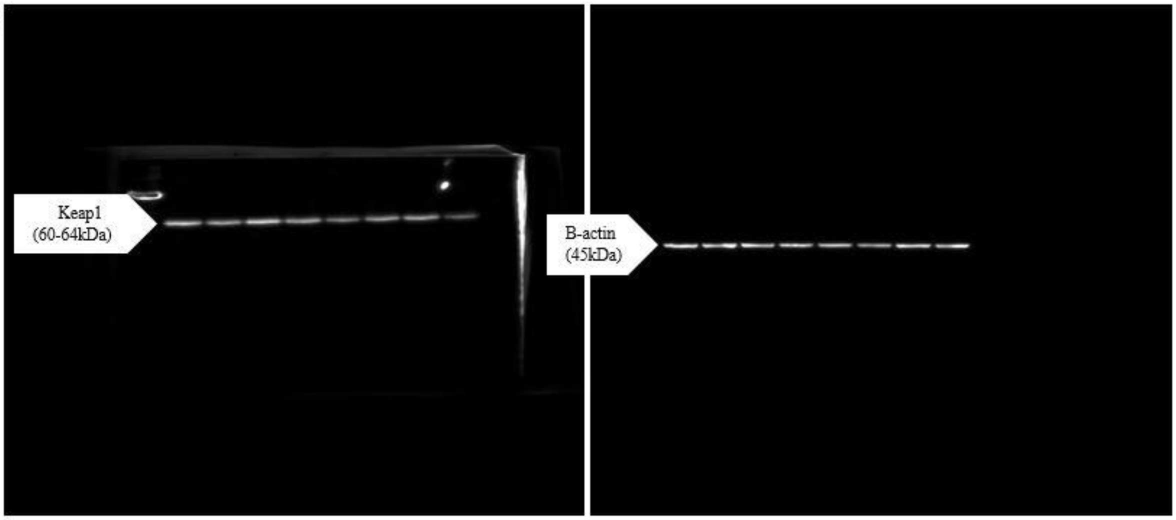

(c)

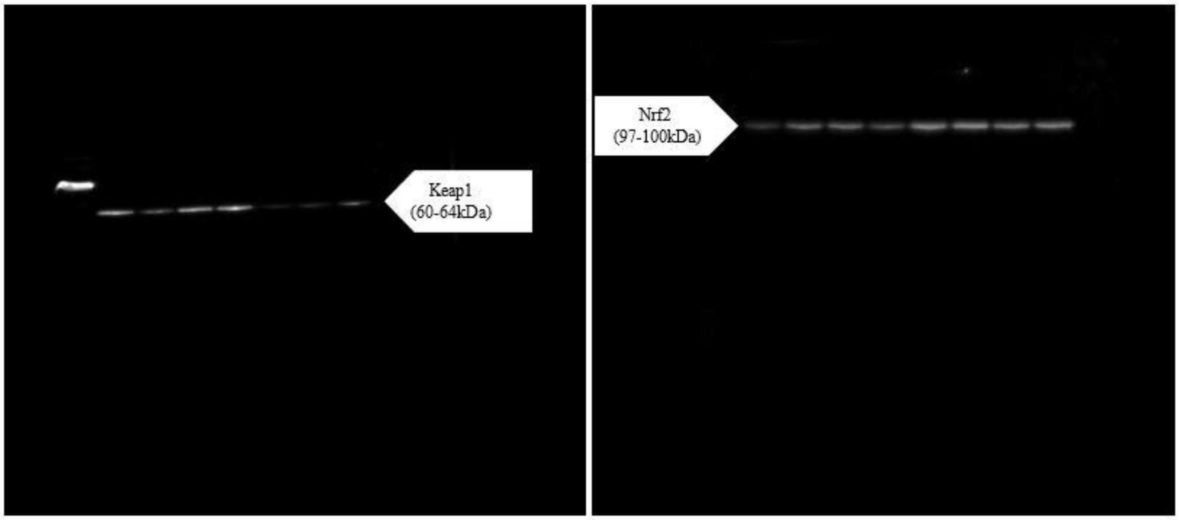

Supplementary Table S1. Effect of the negative control and vehicle control (0.05% DMSO) on the viability of CCD-18Co cells as assessed using MTT cytotoxicity assay. Cells were treated with 0.05% DMSO for 24 hours in parallel with the negative control (culture media with no addition of any treatments). Each data point was obtained from three independent experimental replicates and expressed as mean ± SEM of percentage of cell viability (relative to negative control). SD represents the standard deviation of the mean values between each replicate. SEM represents the standard error of the mean value.

	**Absorbance value at 570nm**			**Percentage of cell viability (%)**					
**Treatment**	**n1**	**n2**	**n3**	**n1**	**n2**	**n3**	**AVE**	**SD**	**SEM**
0.05% DMSO	0.479	0.437	0.475	99.79167	100.2294	100.21097	100.077	0.24756	0.14293
Negative control	0.48	0.436	0.474	100	100	100	100	0	0

remove yellow colour
